# 2-{*N*-[(2,3,4,9-Tetra­hydro-1*H*-carbazol-3-yl)meth­yl]methyl­sulfonamido}­ethyl methane­sulfonate

**DOI:** 10.1107/S1600536813034016

**Published:** 2013-12-21

**Authors:** Mustafa Göçmentürk, Yavuz Ergün, Berline Mougang-Soume, Nagihan Çaylak Delibaş, Tuncer Hökelek

**Affiliations:** a Dokuz Eylül University, Faculty of Arts and Sciences, Department of Chemistry, Tınaztepe, 35160 Buca, İzmir, Turkey; b Université de Montréal, Département de Chimie, H3C 3J7, Montréal, Québec, Canada; c Department of Physics, Sakarya University, 54187 Esentepe, Sakarya, Turkey; d Hacettepe University, Department of Physics, 06800 Beytepe, Ankara, Turkey

## Abstract

In the title compound, C_17_H_24_N_2_O_5_S_2_, the indole ring system is nearly planar [maximum deviation = 0.032 (1) Å] and the cyclo­hexene ring has a half-chair conformation. In the crystal, N—H⋯O hydrogen bonds link the mol­ecules into a chain running along the *b*-axis direction. Weak C—H⋯O hydrogen bonds and weak C—H⋯π inter­actions are observed between the chains.

## Related literature   

For tetra­hydro­carbazole systems present in the framework of a number of indole-type alkaloids of biological inter­est, see: Saxton (1983 ▶). For the anti­tumor activity of tetra­hydro­carbazoles containing an amine unit, see: Chen *et al.* (2009 ▶). For the most potent drugs, such as ellipcitine and olivacine, for the treatment of a variety of cancers, see: Pelletier (1970 ▶). For the use of tetra­hydro­carbazoles in the synthesis of pyridocarbazoles, see: Knölker & Reddy (2002 ▶). For related structures, see: Patır *et al.* (1997 ▶); Gündoğdu *et al.* (2011 ▶); Göçmen­türk *et al.* (2013 ▶).
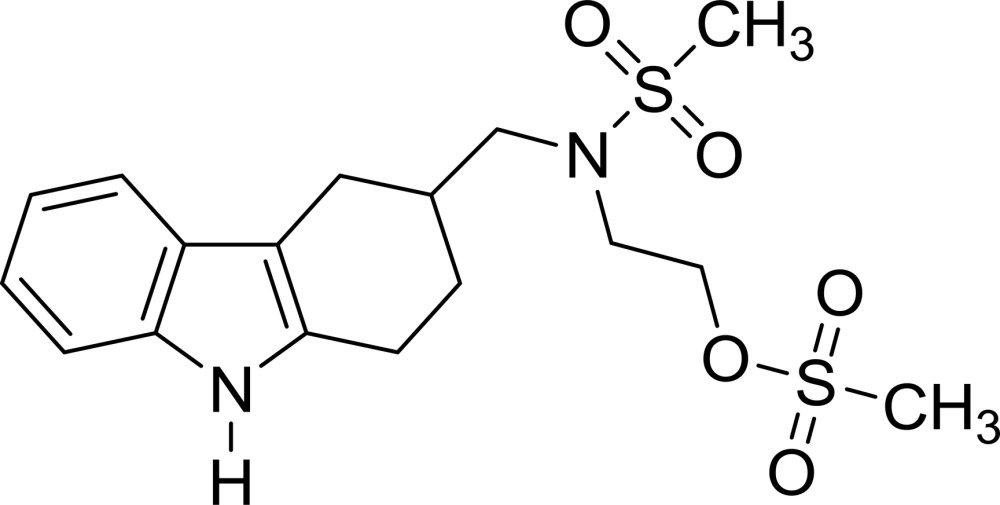


## Experimental   

### 

#### Crystal data  

C_17_H_24_N_2_O_5_S_2_
*M* *_r_* = 400.50
Monoclinic, 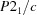

*a* = 5.4399 (2) Å
*b* = 18.0322 (6) Å
*c* = 19.0103 (6) Å
β = 98.973 (2)°
*V* = 1841.96 (11) Å^3^
*Z* = 4
Cu *K*α radiation
μ = 2.90 mm^−1^
*T* = 150 K
0.18 × 0.16 × 0.13 mm

#### Data collection  

Bruker Kappa APEXII CCD area-detector diffractometer
Absorption correction: multi-scan (*SADABS*; Bruker, 2005 ▶) *T*_min_ = 0.623, *T*_max_ = 0.686
47392 measured reflections
3472 independent reflections
3357 reflections with *I* > 2σ(*I*)
*R*_int_ = 0.054

#### Refinement  

*R*[*F* ^2^ > 2σ(*F* ^2^)] = 0.031
*wR*(*F* ^2^) = 0.090
*S* = 1.05
3472 reflections
241 parameters
H atoms treated by a mixture of independent and constrained refinement
Δρ_max_ = 0.41 e Å^−3^
Δρ_min_ = −0.36 e Å^−3^

### 

Data collection: *APEX2* (Bruker, 2007 ▶); cell refinement: *SAINT* (Bruker, 2007 ▶); data reduction: *SAINT*; program(s) used to solve structure: *SHELXS97* (Sheldrick, 2008 ▶); program(s) used to refine structure: *SHELXL97* (Sheldrick, 2008 ▶); molecular graphics: *ORTEP-3 for Windows* (Farrugia, 2012 ▶); software used to prepare material for publication: *WinGX* (Farrugia, 2012 ▶) and *PLATON* (Spek, 2009 ▶).

## Supplementary Material

Crystal structure: contains datablock(s) I, global. DOI: 10.1107/S1600536813034016/xu5758sup1.cif

Structure factors: contains datablock(s) I. DOI: 10.1107/S1600536813034016/xu5758Isup2.hkl

Supporting information file. DOI: 10.1107/S1600536813034016/xu5758Isup3.cml

Additional supporting information:  crystallographic information; 3D view; checkCIF report

## Figures and Tables

**Table 1:** Hydrogen-bond geometry (Å, °) *Cg*2 is the centroid of the C4a/C5a/C8a/N9/C9a ring.

*D*—H⋯*A*	*D*—H	H⋯*A*	*D*⋯*A*	*D*—H⋯*A*
N9—H9⋯O2^i^	0.83 (2)	2.17 (2)	2.9804 (16)	166 (2)
C11—H11*C*⋯O4^ii^	0.98	2.45	3.171 (2)	130
C13—H13*A*⋯O5^iii^	0.99	2.46	3.4148 (19)	161
C14—H14*B*⋯O5^iv^	0.98	2.42	3.317 (2)	152
C11—H11*A*⋯*Cg*2^ii^	0.98	2.95	3.6705 (19)	131

Symmetry codes: (i) 
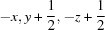
; (ii) 
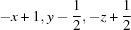
; (iii) 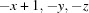; (iv) 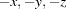.

**Fig. 1.**
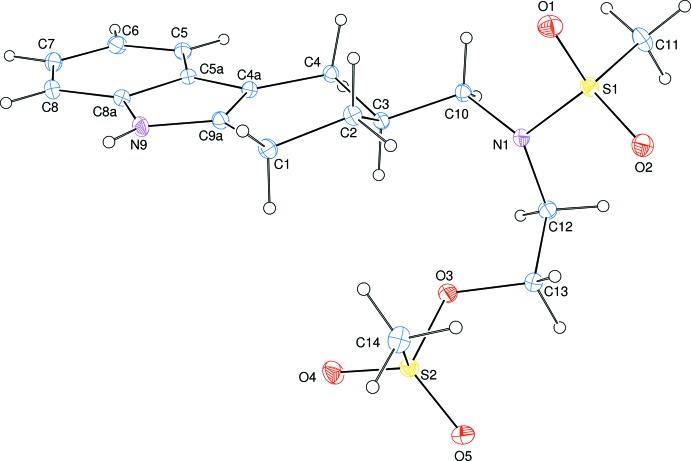
The molecular structure of the title molecule with the atom-numbering scheme. Displacement ellipsoids are drawn at the 50% probability level.

**Fig. 2.**
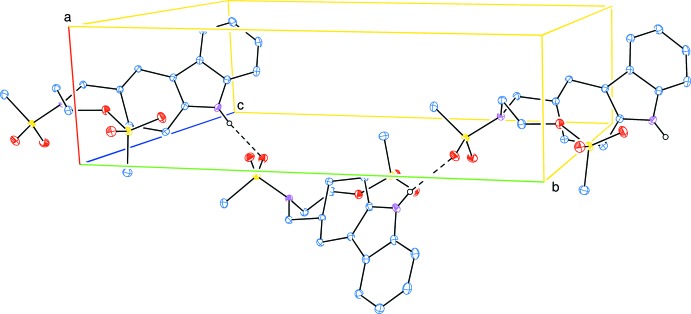
A view of the crystal packing of the title compound. Only the N—H···O hydrogen bonds are shown as dashed lines [H-atoms not involved in hydrogen bonding have been omitted for clarity].

## supplementary crystallographic information

### 1. Comment

Tetrahydrocarbazole systems are present in the framework of a number of indole-type alkaloids of biological interest (Saxton, 1983). The structures of tricyclic, tetracyclic and pentacyclic ring systems with dithiolane and other substituents of the tetrahydrocarbazole core, have been reported previously (Patır et al., 1997). Nitrogen containing heterocyclic compounds are encountered in a very large number of groups of organic compounds. They play a vital role in the metabolism of all living cells, which are widely distributed in nature and are essential to life. One of them pyridocarbazoles such as ellipcitine and olivacine are some of the most potent drugs for the treatment of a variety of cancers (Pelletier, 1970). Tetrahydrocarbazoles have been used as key compounds for the syntheses of various pyridocarbazoles (Knölker & Reddy, 2002). Amine moiety containing tetrahydrocarbazoles have also been showed antitumor activity (Chen et al., 2009). The present study was undertaken to ascertain the crystal structure of the title compound.

The molecule of the title compound contains a carbazole skeleton with methyl sulfonamide and ethyl methanesulfonate groups, (Fig. 1). In all structures atom N9 is substituted.

An examination of the deviations from the least-squares planes through individual rings shows that rings B (C4a/C5a/C8a/N9/C9a) and C (C5a/C5—C8/C8a) are nearly coplanar [with a maximum deviation of 0.032 (1) Å for atom N9] with dihedral angle of B/C = 2.16 (5)°. Ring A (C1—C4/C4a/C9a) adopts half-chair conformation, as in ethyl 4-oxo-2,3,4,9-tetrahydro-1-H-carbazole-3-carboxylate (Gündoğdu et al., 2011) and 2-{4-Methyl-N-[(2,3,4,9-tetrahydro-1H-carbazol-3-yl)methyl]benzenesulfonamido} ethyl 4-methylbenzenesulfonate (Göçmentürk et al., 2013). Ring A has a pseudo twofold axis running through the midpoints of C2–C3 and C4a–C9a bonds.

In the crystal, N—H···O hydrogen bonds (Table 1) link the molecules into a chain running along the b-axis direction (Fig. 2), and weak C—H···O hydrogen bonds and a weak C—H···π interaction (Table 1) are observed between the chains.

### 2. Experimental

For the preparation of the title compound, (I), a solution of 2-((2,3,4,9 -tetrahydro-1*H*-carbazole-3-yl)methylamino)ethanol (1.0 g, 4.1 mmol) in pyridine (5 ml) was cooled to 273 K. Then, methanesulphonyl chloride (1.0 g, 9.0 mmol) was added dropwise. The mixture was stirred for 18 h at room temperature, and then washed with hydrochloric acid (10%). The organic layer was extracted with chloroform and dried over anhydrous magnesium sulfate. The solvent was removed under reduced pressure. The crude product was purified by silica gel column chromatography eluting with ethyl acetate:hexane (1:1). The solvent was evaporated under reduced pressure and the residue was recrystallized from methanol (yield; 1.1 g, 67%, m.p. 404 K).

### 3. Refinement

H9 atom is located in a difference Fourier synthesis and refined isotropically. The remaining C-bound H-atoms were positioned geometrically with C—H = 0.95, 1.00, 0.99 and 0.98 Å, for aromatic, methine, methylene and methyl H-atoms, respectively, and constrained to ride on their parent atoms, with *U*_iso_(H) = k × *U*_eq_(C), where k = 1.5 for methyl H-atoms and k = 1.2 for all other H-atoms.

### Crystal data

**Table:**

C_17_H_24_N_2_O_5_S_2_	*F*(000) = 848
*M**_r_* = 400.50	*D*_x_ = 1.444 Mg m^−^^3^
Monoclinic, *P*2_1_/*c*	Cu *K*α radiation, λ = 1.54178 Å
Hall symbol: -P 2ybc	Cell parameters from 9016 reflections
*a* = 5.4399 (2) Å	θ = 3.4–69.5°
*b* = 18.0322 (6) Å	µ = 2.90 mm^−^^1^
*c* = 19.0103 (6) Å	*T* = 150 K
β = 98.973 (2)°	Plate, colourless
*V* = 1841.96 (11) Å^3^	0.18 × 0.16 × 0.13 mm
*Z* = 4	

### Data collection

**Table:**

Bruker Kappa APEXII CCD area-detector diffractometer	3472 independent reflections
Radiation source: fine-focus sealed tube	3357 reflections with *I* > 2σ(*I*)
Graphite monochromator	*R*_int_ = 0.054
φ and ω scans	θ_max_ = 69.8°, θ_min_ = 3.4°
Absorption correction: multi-scan (*SADABS*; Bruker, 2005)	*h* = −6→6
*T*_min_ = 0.623, *T*_max_ = 0.686	*k* = −20→21
47392 measured reflections	*l* = −23→23

### Refinement

**Table:**

Refinement on *F*^2^	Primary atom site location: structure-invariant direct methods
Least-squares matrix: full	Secondary atom site location: difference Fourier map
*R*[*F*^2^ > 2σ(*F*^2^)] = 0.031	Hydrogen site location: inferred from neighbouring sites
*wR*(*F*^2^) = 0.090	H atoms treated by a mixture of independent and constrained refinement
*S* = 1.05	*w* = 1/[σ^2^(*F*_o_^2^) + (0.0569*P*)^2^ + 0.6777*P*] where *P* = (*F*_o_^2^ + 2*F*_c_^2^)/3
3472 reflections	(Δ/σ)_max_ < 0.001
241 parameters	Δρ_max_ = 0.41 e Å^−^^3^
0 restraints	Δρ_min_ = −0.36 e Å^−^^3^

### Special details

**Table:**

Geometry. All esds (except the esd in the dihedral angle between two l.s. planes) are estimated using the full covariance matrix. The cell esds are taken into account individually in the estimation of esds in distances, angles and torsion angles; correlations between esds in cell parameters are only used when they are defined by crystal symmetry. An approximate (isotropic) treatment of cell esds is used for estimating esds involving l.s. planes.
Refinement. Refinement of F^2^ against ALL reflections. The weighted R-factor wR and goodness of fit S are based on F^2^, conventional R-factors R are based on F, with F set to zero for negative F^2^. The threshold expression of F^2^ > 2sigma(F^2^) is used only for calculating R-factors(gt) etc. and is not relevant to the choice of reflections for refinement. R-factors based on F^2^ are statistically about twice as large as those based on F, and R- factors based on ALL data will be even larger.

### Fractional atomic coordinates and isotropic or equivalent isotropic displacement parameters (Å^2^)

**Table:**

	*x*	*y*	*z*	*U*_iso_*/*U*_eq_	
S1	0.19263 (6)	−0.204476 (18)	0.209478 (18)	0.02367 (12)	
S2	0.21420 (6)	0.077279 (19)	0.091542 (18)	0.02505 (12)	
O1	0.0230 (2)	−0.20118 (6)	0.25961 (6)	0.0344 (3)	
O2	0.0933 (2)	−0.21216 (6)	0.13524 (6)	0.0339 (3)	
O3	0.34759 (19)	0.01082 (6)	0.13520 (5)	0.0276 (2)	
O4	0.3080 (2)	0.14168 (6)	0.12962 (6)	0.0368 (3)	
O5	0.2426 (2)	0.06975 (6)	0.01834 (6)	0.0328 (3)	
N1	0.3567 (2)	−0.12840 (6)	0.21645 (6)	0.0218 (2)	
N9	0.2800 (2)	0.16928 (7)	0.40969 (7)	0.0264 (3)	
H9	0.161 (4)	0.1983 (11)	0.4018 (10)	0.035 (5)*	
C1	0.1172 (3)	0.07536 (8)	0.31395 (8)	0.0252 (3)	
H1A	0.1370	0.1042	0.2709	0.030*	
H1B	−0.0549	0.0824	0.3236	0.030*	
C2	0.1640 (2)	−0.00717 (8)	0.30133 (8)	0.0243 (3)	
H2A	0.0927	−0.0369	0.3370	0.029*	
H2B	0.0768	−0.0213	0.2536	0.029*	
C3	0.4407 (2)	−0.02574 (7)	0.30626 (7)	0.0210 (3)	
H3	0.5135	0.0055	0.2713	0.025*	
C4	0.5787 (2)	−0.00923 (7)	0.38158 (7)	0.0215 (3)	
H4A	0.5346	−0.0471	0.4152	0.026*	
H4B	0.7606	−0.0117	0.3817	0.026*	
C4A	0.5100 (3)	0.06626 (7)	0.40525 (7)	0.0217 (3)	
C5	0.8579 (3)	0.10872 (8)	0.50849 (8)	0.0265 (3)	
H5	0.9663	0.0676	0.5071	0.032*	
C5A	0.6340 (3)	0.11314 (7)	0.46082 (7)	0.0229 (3)	
C6	0.9185 (3)	0.16495 (9)	0.55752 (8)	0.0315 (3)	
H6	1.0704	0.1624	0.5898	0.038*	
C7	0.7590 (3)	0.22570 (9)	0.56039 (8)	0.0338 (4)	
H7	0.8029	0.2628	0.5956	0.041*	
C8	0.5396 (3)	0.23279 (8)	0.51321 (8)	0.0318 (3)	
H8	0.4330	0.2743	0.5149	0.038*	
C8A	0.4810 (3)	0.17645 (8)	0.46293 (7)	0.0253 (3)	
C9A	0.2978 (3)	0.10205 (8)	0.37574 (7)	0.0233 (3)	
C10	0.4772 (3)	−0.10741 (7)	0.28916 (7)	0.0228 (3)	
H10A	0.6576	−0.1180	0.2940	0.027*	
H10B	0.4078	−0.1384	0.3243	0.027*	
C11	0.4043 (3)	−0.27709 (9)	0.23392 (10)	0.0360 (4)	
H11A	0.5265	−0.2786	0.2011	0.054*	
H11B	0.3142	−0.3243	0.2317	0.054*	
H11C	0.4902	−0.2689	0.2826	0.054*	
C12	0.4941 (3)	−0.11271 (8)	0.15714 (8)	0.0251 (3)	
H12A	0.5300	−0.1600	0.1344	0.030*	
H12B	0.6551	−0.0892	0.1762	0.030*	
C13	0.3519 (3)	−0.06250 (8)	0.10170 (8)	0.0264 (3)	
H13A	0.4356	−0.0598	0.0591	0.032*	
H13B	0.1804	−0.0812	0.0871	0.032*	
C14	−0.0999 (3)	0.06534 (9)	0.09937 (9)	0.0344 (4)	
H14B	−0.1577	0.0172	0.0792	0.052*	
H14A	−0.1987	0.1050	0.0735	0.052*	
H14C	−0.1191	0.0670	0.1498	0.052*	

### Atomic displacement parameters (Å^2^)

**Table:**

	*U*^11^	*U*^22^	*U*^33^	*U*^12^	*U*^13^	*U*^23^
S1	0.02243 (19)	0.02128 (19)	0.0264 (2)	−0.00275 (12)	0.00095 (14)	−0.00032 (12)
S2	0.0270 (2)	0.0233 (2)	0.0241 (2)	−0.00371 (12)	0.00149 (14)	0.00101 (12)
O1	0.0284 (5)	0.0383 (6)	0.0378 (6)	−0.0057 (4)	0.0092 (5)	0.0024 (5)
O2	0.0369 (6)	0.0322 (6)	0.0296 (6)	−0.0095 (5)	−0.0040 (5)	−0.0024 (4)
O3	0.0334 (6)	0.0244 (5)	0.0238 (5)	−0.0008 (4)	0.0004 (4)	0.0004 (4)
O4	0.0429 (6)	0.0259 (6)	0.0385 (6)	−0.0081 (5)	−0.0030 (5)	−0.0022 (5)
O5	0.0395 (6)	0.0337 (6)	0.0256 (6)	−0.0008 (5)	0.0062 (5)	0.0050 (4)
N1	0.0236 (6)	0.0200 (6)	0.0217 (6)	−0.0010 (4)	0.0029 (4)	−0.0018 (4)
N9	0.0280 (6)	0.0215 (6)	0.0297 (7)	0.0076 (5)	0.0042 (5)	−0.0005 (5)
C1	0.0230 (7)	0.0234 (7)	0.0287 (7)	0.0035 (5)	0.0021 (6)	0.0012 (5)
C2	0.0210 (6)	0.0231 (7)	0.0283 (7)	0.0006 (5)	0.0020 (5)	−0.0005 (5)
C3	0.0216 (6)	0.0190 (6)	0.0227 (7)	0.0001 (5)	0.0039 (5)	−0.0003 (5)
C4	0.0212 (6)	0.0207 (6)	0.0226 (7)	0.0024 (5)	0.0032 (5)	−0.0003 (5)
C4A	0.0237 (6)	0.0202 (6)	0.0218 (7)	0.0015 (5)	0.0059 (5)	0.0002 (5)
C5	0.0295 (7)	0.0254 (7)	0.0241 (7)	0.0012 (6)	0.0026 (6)	0.0006 (5)
C5A	0.0276 (7)	0.0212 (7)	0.0207 (6)	0.0000 (5)	0.0065 (5)	0.0010 (5)
C6	0.0363 (8)	0.0309 (8)	0.0255 (7)	−0.0042 (6)	−0.0008 (6)	−0.0002 (6)
C7	0.0492 (9)	0.0253 (7)	0.0262 (7)	−0.0048 (7)	0.0045 (7)	−0.0061 (6)
C8	0.0442 (9)	0.0218 (7)	0.0304 (8)	0.0029 (6)	0.0090 (7)	−0.0024 (6)
C8A	0.0317 (7)	0.0218 (7)	0.0235 (7)	0.0018 (6)	0.0073 (6)	0.0013 (5)
C9A	0.0246 (7)	0.0210 (7)	0.0251 (7)	0.0019 (5)	0.0062 (5)	0.0009 (5)
C10	0.0241 (7)	0.0209 (7)	0.0224 (7)	0.0014 (5)	0.0004 (5)	−0.0013 (5)
C11	0.0378 (9)	0.0207 (7)	0.0474 (10)	0.0012 (6)	0.0001 (7)	0.0010 (7)
C12	0.0243 (7)	0.0255 (7)	0.0262 (7)	0.0000 (5)	0.0062 (6)	−0.0001 (5)
C13	0.0310 (7)	0.0244 (7)	0.0239 (7)	−0.0016 (6)	0.0047 (6)	−0.0016 (5)
C14	0.0279 (8)	0.0381 (8)	0.0371 (9)	−0.0018 (6)	0.0049 (6)	−0.0067 (7)

### Geometric parameters (Å, º)

**Table:**

S1—O1	1.4273 (12)	C4A—C9A	1.365 (2)
S1—O2	1.4370 (11)	C5—C6	1.382 (2)
S1—N1	1.6306 (12)	C5—H5	0.9500
S1—C11	1.7576 (16)	C5A—C4A	1.4363 (19)
S2—O3	1.5697 (10)	C5A—C5	1.402 (2)
S2—O4	1.4205 (11)	C5A—C8A	1.4169 (19)
S2—O5	1.4303 (11)	C6—C7	1.404 (2)
S2—C14	1.7516 (16)	C6—H6	0.9500
O3—C13	1.4693 (17)	C7—C8	1.383 (2)
N1—C10	1.4836 (17)	C7—H7	0.9500
N1—C12	1.4742 (18)	C8—C8A	1.397 (2)
N9—C8A	1.3753 (19)	C8—H8	0.9500
N9—C9A	1.3837 (18)	C9A—C1	1.489 (2)
N9—H9	0.83 (2)	C10—H10A	0.9900
C1—C2	1.5350 (19)	C10—H10B	0.9900
C1—H1A	0.9900	C11—H11A	0.9800
C1—H1B	0.9900	C11—H11B	0.9800
C2—C3	1.5304 (18)	C11—H11C	0.9800
C2—H2A	0.9900	C12—C13	1.508 (2)
C2—H2B	0.9900	C12—H12A	0.9900
C3—C4	1.5386 (18)	C12—H12B	0.9900
C3—C10	1.5278 (18)	C13—H13A	0.9900
C3—H3	1.0000	C13—H13B	0.9900
C4—H4A	0.9900	C14—H14B	0.9800
C4—H4B	0.9900	C14—H14A	0.9800
C4A—C4	1.4992 (18)	C14—H14C	0.9800
			
O1—S1—O2	118.47 (7)	C5—C5A—C4A	134.64 (13)
O1—S1—N1	108.26 (6)	C5—C5A—C8A	118.82 (13)
O1—S1—C11	108.63 (8)	C8A—C5A—C4A	106.54 (12)
O2—S1—N1	106.15 (6)	C5—C6—C7	120.98 (14)
O2—S1—C11	108.56 (8)	C5—C6—H6	119.5
N1—S1—C11	106.12 (7)	C7—C6—H6	119.5
O3—S2—C14	103.76 (7)	C6—C7—H7	119.2
O4—S2—O3	104.78 (6)	C8—C7—C6	121.55 (14)
O4—S2—O5	119.25 (7)	C8—C7—H7	119.2
O4—S2—C14	109.56 (8)	C7—C8—C8A	117.27 (14)
O5—S2—O3	109.32 (6)	C7—C8—H8	121.4
O5—S2—C14	109.02 (8)	C8A—C8—H8	121.4
C13—O3—S2	119.68 (9)	N9—C8A—C5A	107.83 (12)
C10—N1—S1	116.55 (9)	N9—C8A—C8	129.95 (14)
C12—N1—S1	115.83 (9)	C8—C8A—C5A	122.21 (14)
C12—N1—C10	117.43 (11)	N9—C9A—C1	124.46 (12)
C8A—N9—C9A	108.69 (12)	C4A—C9A—N9	109.80 (13)
C8A—N9—H9	125.7 (13)	C4A—C9A—C1	125.68 (13)
C9A—N9—H9	125.3 (13)	N1—C10—C3	113.02 (11)
C2—C1—H1A	109.8	N1—C10—H10A	109.0
C2—C1—H1B	109.8	N1—C10—H10B	109.0
C9A—C1—C2	109.37 (11)	C3—C10—H10A	109.0
C9A—C1—H1A	109.8	C3—C10—H10B	109.0
C9A—C1—H1B	109.8	H10A—C10—H10B	107.8
H1A—C1—H1B	108.2	S1—C11—H11A	109.5
C1—C2—H2A	109.0	S1—C11—H11B	109.5
C1—C2—H2B	109.0	S1—C11—H11C	109.5
C3—C2—C1	112.84 (11)	H11A—C11—H11B	109.5
C3—C2—H2A	109.0	H11A—C11—H11C	109.5
C3—C2—H2B	109.0	H11B—C11—H11C	109.5
H2A—C2—H2B	107.8	N1—C12—C13	112.57 (11)
C2—C3—C4	110.32 (11)	N1—C12—H12A	109.1
C2—C3—H3	108.9	N1—C12—H12B	109.1
C4—C3—H3	108.9	C13—C12—H12A	109.1
C10—C3—C2	110.92 (11)	C13—C12—H12B	109.1
C10—C3—C4	108.89 (11)	H12A—C12—H12B	107.8
C10—C3—H3	108.9	O3—C13—C12	106.16 (11)
C3—C4—H4A	109.6	O3—C13—H13A	110.5
C3—C4—H4B	109.6	O3—C13—H13B	110.5
C4A—C4—C3	110.28 (11)	C12—C13—H13A	110.5
C4A—C4—H4A	109.6	C12—C13—H13B	110.5
C4A—C4—H4B	109.6	H13A—C13—H13B	108.7
H4A—C4—H4B	108.1	S2—C14—H14B	109.5
C5A—C4A—C4	130.15 (13)	S2—C14—H14A	109.5
C9A—C4A—C5A	107.10 (12)	S2—C14—H14C	109.5
C9A—C4A—C4	122.73 (13)	H14B—C14—H14A	109.5
C5A—C5—H5	120.5	H14B—C14—H14C	109.5
C6—C5—C5A	119.09 (14)	H14A—C14—H14C	109.5
C6—C5—H5	120.5		
			
O1—S1—N1—C10	50.95 (11)	C5A—C4A—C4—C3	−161.46 (13)
O1—S1—N1—C12	−164.56 (10)	C9A—C4A—C4—C3	20.88 (18)
O2—S1—N1—C10	179.12 (10)	C4—C4A—C9A—N9	178.07 (12)
O2—S1—N1—C12	−36.39 (11)	C4—C4A—C9A—C1	−4.8 (2)
C11—S1—N1—C10	−65.50 (12)	C5A—C4A—C9A—N9	−0.06 (16)
C11—S1—N1—C12	78.99 (12)	C5A—C4A—C9A—C1	177.07 (13)
O4—S2—O3—C13	−162.57 (10)	C5A—C5—C6—C7	0.4 (2)
O5—S2—O3—C13	−33.66 (12)	C5—C5A—C4A—C4	3.9 (3)
C14—S2—O3—C13	82.53 (11)	C5—C5A—C4A—C9A	−178.12 (16)
S2—O3—C13—C12	179.57 (9)	C8A—C5A—C4A—C4	−176.65 (13)
S1—N1—C10—C3	−133.41 (10)	C8A—C5A—C4A—C9A	1.29 (15)
C12—N1—C10—C3	82.67 (14)	C4A—C5A—C5—C6	−178.55 (15)
S1—N1—C12—C13	94.59 (12)	C8A—C5A—C5—C6	2.1 (2)
C10—N1—C12—C13	−121.24 (13)	C4A—C5A—C8A—N9	−2.05 (15)
C9A—N9—C8A—C5A	2.05 (16)	C4A—C5A—C8A—C8	177.19 (13)
C9A—N9—C8A—C8	−177.12 (15)	C5—C5A—C8A—N9	177.48 (13)
C8A—N9—C9A—C1	−178.42 (13)	C5—C5A—C8A—C8	−3.3 (2)
C8A—N9—C9A—C4A	−1.25 (16)	C5—C6—C7—C8	−1.9 (2)
C9A—C1—C2—C3	−43.77 (16)	C6—C7—C8—C8A	0.8 (2)
C1—C2—C3—C4	62.50 (15)	C7—C8—C8A—N9	−179.12 (15)
C1—C2—C3—C10	−176.76 (11)	C7—C8—C8A—C5A	1.8 (2)
C2—C3—C4—C4A	−47.92 (15)	N9—C9A—C1—C2	−167.67 (13)
C10—C3—C4—C4A	−169.87 (11)	C4A—C9A—C1—C2	15.6 (2)
C2—C3—C10—N1	60.12 (15)	N1—C12—C13—O3	69.55 (14)
C4—C3—C10—N1	−178.29 (11)		

### Hydrogen-bond geometry (Å, º)

Cg2 is the centroid of the C4a/C5a/C8a/N9/C9a ring.

**Table:**

*D*—H···*A*	*D*—H	H···*A*	*D*···*A*	*D*—H···*A*
N9—H9···O2^i^	0.83 (2)	2.17 (2)	2.9804 (16)	166 (2)
C11—H11*C*···O4^ii^	0.98	2.45	3.171 (2)	130
C13—H13*A*···O5^iii^	0.99	2.46	3.4148 (19)	161
C14—H14*B*···O5^iv^	0.98	2.42	3.317 (2)	152
C11—H11*A*···*Cg*2^ii^	0.98	2.95	3.6705 (19)	131

Symmetry codes: (i) −*x*, *y*+1/2, −*z*+1/2; (ii) −*x*+1, *y*−1/2, −*z*+1/2; (iii) −*x*+1, −*y*, −*z*; (iv) −*x*, −*y*, −*z*.
